# Bone marrow aspirate and bone marrow aspirate concentrate: Does the literature support use in long-bone nonunion and provide new insights into mechanism of action?

**DOI:** 10.1007/s00590-024-04048-9

**Published:** 2024-07-26

**Authors:** Andrew J. Moyal, Austin W. Li, Jeremy M. Adelstein, Tyler J. Moon, Joshua K. Napora

**Affiliations:** 1 University Hospitals Cleveland Medical Center, 11100 Euclid Ave, Cleveland, OH 44106 USA; 2 Case Western Reserve University School of Medicine, 10900 Euclid Ave, Cleveland, OH 44106 USA; 3 Department of Orthopedic Surgery, University Hospitals Cleveland Medical Center, Case Western Reserve University, 11100 Euclid Ave, Cleveland, OH 44106 USA

**Keywords:** Nonunion, Long bones, Mesenchymal stem cells, Bone marrow, Bone marrow aspirate concentration

## Abstract

**Purpose:**

To assess the use of bone marrow aspirate (BM) and bone marrow aspirate concentrate (BMAC) in the treatment of long-bone nonunion and to understand mechanism of action.

**Methods:**

A systematic review of PubMed and EBSCOHost was completed to identify studies that investigated the use of BM or BMAC for the diagnosis of delayed union and/or nonunion of long-bone fractures. Studies of isolated bone marrow-mesenchymal stem cells (BM-MSCs) and use in non-long-bone fractures were excluded. Statistical analysis was confounded by heterogeneous fracture fixation methods, treatment history, and scaffold use.

**Results:**

Our initial search yielded 430 publications, which was screened down to 25 studies. Successful treatment in aseptic nonunion was reported at 79–100% (BM) and 50–100% (BMAC). Septic nonunion rates were slightly better at 73–100% (BM) and 83.3–100% (BMAC). 18/24 studies report union rates > 80%. One study reports successful treatment of septic nonunion with BMAC and no antibiotics. A separate study reported a significant reduction in autograft reinfection rate when combined with BMAC (*P* = 0.009). Major adverse events include two deep infections at injection site and one case of heterotopic ossification. Most studies note transient mild donor site discomfort and potential injection site discomfort attributed to needle size.

**Conclusion:**

The current literature pertaining to use of BM/BMAC for nonunion is extremely heterogeneous in terms of patient population and concomitant treatment modalities. While results are promising for use of BM/BMAC with other gold standard treatment methodologies, the literature requires additional Level I data to clarify the impact of role BM/BMAC in treating nonunion when used alone and in combination with other modalities.

**Level of evidence:**

Level III.

## Introduction

Successful treatment of nonunion is predicated on four major tenants: requirement of an osteoconductive scaffold for new bone formation, adequate delivery of osteogenic cells, an osteoinductive pro-growth signaling environment, and mechanical stability at the fracture site [1]. The relative importance of these factors differs depending on etiology of nonunion (atrophic, oligotrophic, or hypertrophic) and presence of infection, but successful treatment requires all components [2, 3]. Traditional surgical modalities and bone grafting successfully address the requirements for mechanical stability and osteoconductive scaffold. Fresh autologous bone graft is the gold standard for nonunion treatment as it provides the osteogenic cells, osteoinductive growth factors, and osteoconductive scaffold required for new bone formation [4]. However, the treatment suffers from the drawbacks of required reoperation, limited supply, and patient morbidity including pain and infection risk [4, 5].

Research has focused on finding less invasive therapies with osteogenic and osteoinductive potential [6]. Bone marrow aspirate (BM) and bone marrow aspirate concentrate (BMAC) are among two of the more popular FDA approved adjuvants used to specifically addressing these limitations [6, 7]. BM and BMAC deliver the necessary progenitor cells and pro-growth signaling molecules required for osteogenesis and osteoinduction [8]. While BM simply isolates and re-injects the bone marrow, BMAC takes the added step of spinning down larger volumes of pure bone marrow aspirate in order to increase the overall numbers of injected cells [9]. Importantly, these therapeutics deliver a heterogeneous population of cells, including granulocytes and macrophages, which may specifically aid in treatment of septic nonunion [10].

While early clinical trials and reviews highlight promising results in the treatment of nonunion, none currently compare between use in aseptic vs septic nonunion. Furthermore, given BM-based treatments are around 0.001–0.01% pluripotent cells, no reports definitively outline a mechanism of action for BM-based therapeutics [11]. The aim of this review is to answer the following main questions: (1) Is use of BM and BMAC supported in the treatment of long-bone nonunion, with subsequent attention paid to mechanism of nonunion? (2). Does recent literature highlight a potential mechanism of action?

## Methods

English-language studies were identified through a systematic review conducted on June 15th, using PubMed (National Library of Medicine) and EBSCOhost (MEDLINE with Full Text). Search term focused on the diagnosis of delayed and/or nonunion in order to account for differences in definition based on time [12]. Medical Subject Heading (MeSH) terms were used to capture the following terms: Bone Marrow Transplantation, BMAC, Bone Marrow Cells, Fractures Ununited, Fracture Nonunion, Delayed Union, Fracture Healing, Delayed Union, Fracture Nonunion. Level of evidence was assessed based on guidelines set forth by The Journal of Bone and Joint Surgery [13]. Literature search was performed by three independent authors (AL, AM, and JA). When authors disagreed on inclusion, senior author (JN) was used to make the final decision.

- *Question 1*: Is use of BM and BMAC supported in the treatment of long-bone aseptic and septic nonunion?

Studies were included based on the following criteria. Inclusion criteria: Studies including use of BM and/or BMAC in human subjects with diagnosed nonunion or delayed union. Studies needed to specifically mention septic vs aseptic nonunion, use in long-bone fractures, BM versus BMAC, scaffold use, and union rate. Studies were excluded for no full-length English manuscript available, review articles, use in non-long-bone fractures, diagnosis other than delayed union/nonunion (congenital pseudoarthrosis, atypical fracture, bone defect), and any manipulation of bone marrow that would fall outside the “minimally manipulated” stipulation set forth by the FDA. Minimal manipulation is defined as any process that alters the relevant biologic characteristics of cells and tissues [14]. Pertaining to this last criteria, any manipulation that changes the content of bone aspirate, such is isolating mesenchymal stem cells or any other cell, is currently not FDA approved for use in the USA.

Studies were stratified into use for septic nonunion, use for aseptic nonunion, and mixed use case. Important outcomes identified included union rate, adverse events, and eradication of infection when applicable. No statistical analysis was performed.

- *Question 2: Does recent literature highlight a potential mechanism of action?*

Upon reviewing all titles and abstracts for question 1, articles which included discussion of potential mechanism of action were marked for further analysis. Given the potential mechanism of BM/BMAC, emphasis was placed on highlighting studies which indicate BM and BMACs ability to function via direct differentiation into needed cell lines and/or signaling and improving endogenous cell ability to heal a fracture.

## Results

- *Question 1. Is use of BM and BMAC supported in the treatment of long-bone aseptic and septic nonunion?*

### Study selection

**Fig. 1:**
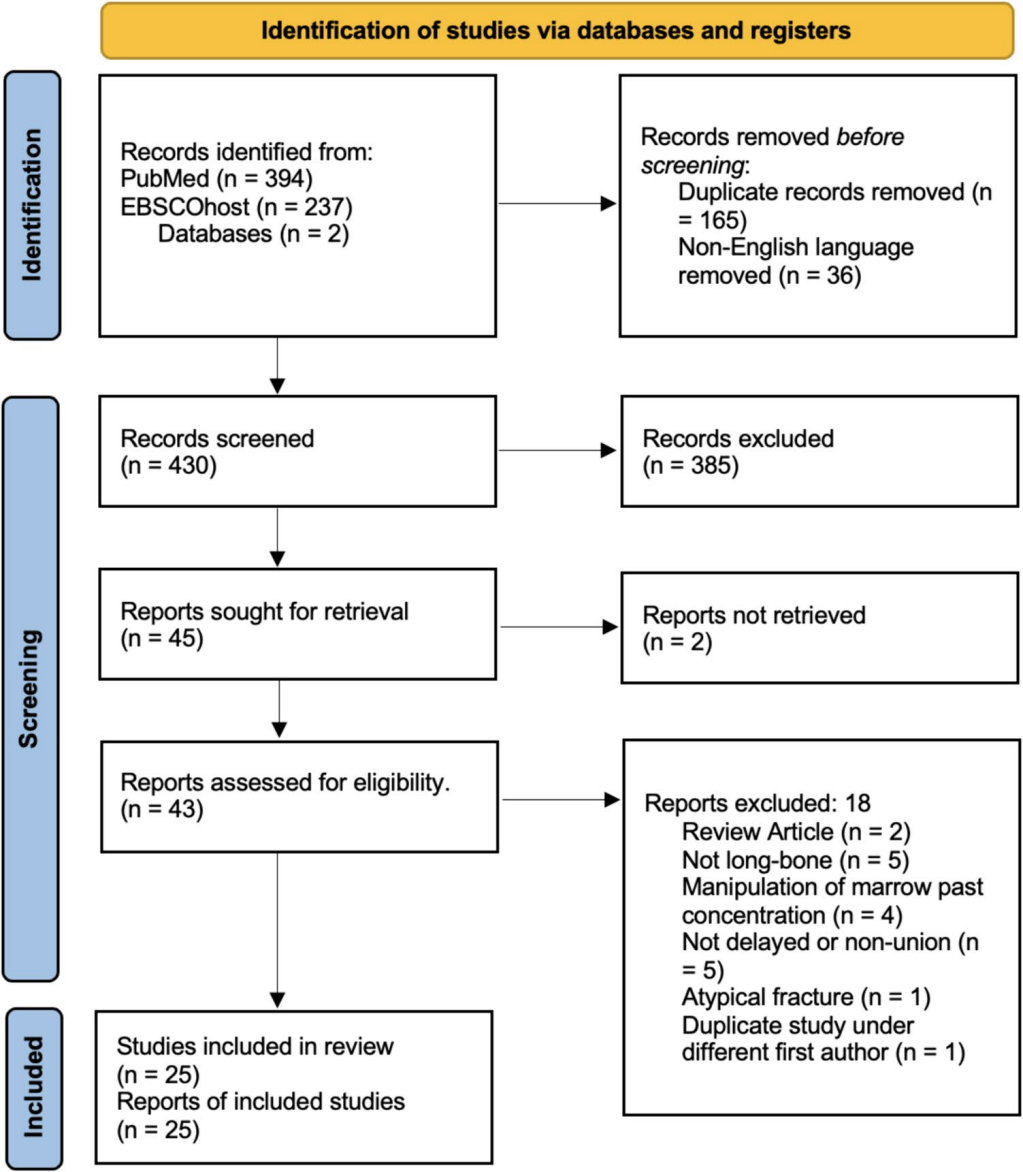
PRISMA flow diagram for review of bone marrow (BM) and bone marrow aspirate concentrate (BMAC) in treatment of nonunion

Literature search provided 631 articles for review. Following de-duplication and screening for full-length English manuscripts, 430 articles were left for independent review by 3 co-authors. Through screening of title and abstracts, 45 articles were identified for retrieval and in-depth analysis. Two articles could not be retrieved. Of the final 43 articles, 18 were excluded according to the PRISMA flow chart (Fig. 1).

### Aseptic nonunion

Twelve studies report on the use of BM or BMAC for the treatment of aseptic nonunion [2, 15–25]. Published articles ranged from 1990 to 2018, with one study pertaining to use in pediatric patients [25]. Level of evidence ranged from IV to II. Six studies report on BM and six report on BMAC. The most common site of nonunion treated was tibia, followed by femur, humerus, and radius/ulna. Seven studies reported on use of BM or BMAC alone, while five studies included use of scaffold [2, 16, 17, 20, 25]. Scaffolds included porous collagen + bovine fibrillar collagen [16], demineralized bone matrix (DBM) or DBM composite (3) [2, 17, 25], or allogenic graft [20]. Study populations ranged from 5 to 66 patients. See Table 1 for an overview of aseptic nonunions including biologic + scaffold combinations.

**Table 1:** Overview of studies utilizing bone marrow (BM) and bone marrow aspirate concentrate (BMAC) for treatment of aseptic nonunion

	Study size	Location	Biologic	Volume	Scaffold	Union rate	Level of evidence
Kocialkowski, 1990 [16]	11	Humerus (2), radius (1), femur (4), tibia (2), rearthrodesis (NA—2)	BM	15–25 mL total volume with scaffold	Porous calcium phosphate + bovine fibrillar cartilage	1	IV
Wilkins, 2003 [17]	66 patients, 69 grafts	Hum (4), ulna (2), radius (2), femur (16), tibia (36), fibula (1), ankle (6—NA)	BM	Not reported	Allogenic dbm composite	0.88 with one treatment, .99 with repeat procedure for non-responders	II
Goel, 2005 [19]	20	All tibia: hypertrophic (10), atrophic (10)	BM	15 mL BM q6 weeks: 1 injection (2), 2 injections (9), 3 injections (8)	None	0.79 (1 loss to follow-up)	II
Braly, 2013 [21]	11	Tibia	BM	40–80 mL	None	0.82	III
Singh, 2013 [22]	12 patients, 11 long bone	Humerus(2), ulna (6), femur (3), metacarpal (NA—1)	BM	30–40 mL every 6 weeks for 2–3 injections	None	0.82	III
Wu, 2018 [26]	53 (Peds 3—18yo)	Humerus (10), radius (5), ulna (5), femur (20), tibia (12), fibula (1)	BM	12.7 mL (repeat every 1–2 mo up to 3 times)	None	0.89	II
Hernigou, 2005 [18]	60	Tibia	BMAC	20 mL BMAC (2579 progenitors/mL–51E3 CFUs)	None	0.88	III
Garnavos, 2010 [2]	5	Humerus	BMAC	10 mL BMAC	DBM	1	III
Vulcano, 2012 [20]	10 patients, 8 nonunion	Nonunion + bone defect: humerus (1), femur (6), tibia (1). 2 patients not diagnosed with nonunion/delayed union	BMAC	10 mL	Allogenic bone graft	1.00 in nonunion, .900 overall	II
Guimares, 2014 [23]	16	Femur	BMAC	40 mL	None	0.5	II
Sugaya, 2014 [24]	17 cases (16 patients)	Humerus (1), Ulna (1), Femur (10), Tibia (5)	BMAC	30–40 mL	None	0.76	III
Desai, 2015 [25]	49, 20 on bisphosphonates	Humerus (7), femur (19), tibia/fibula (23)	BMAC	10 mL	DBM vs DBM + rhBMP-2	.864 (no BMP) vs 0.708 (with BMP)	II

Timing of application of BM of BMAC ranged from 2.5 to 97mo from date of original injury. Volume of BM used ranged from 12.7 (pediatric study) to 80 mL, with 30–40 mL being the most common dose. BMAC was injected at a volume of between 10 and 40 mL and uniformly required 60–120 mL of aspirate to obtain the needed volume of concentrate. While most studies performed one injection, 3 studies utilized repeat injections with continued nonunion on follow-up imaging [19, 21, 26].

Three studies report union rates below 80%, one using BM (79%) and two using BMAC (50%, 76%) [19, 23, 24]. All other articles reported union rates of 82–100% (Table 1). In cases where repeat injections were performed for continued nonunion of the treated site, union rates were subsequently 79–89% [19, 21, 26]. Time to union varied widely, with some studies reporting union in as early as 4 weeks for individuals [22] and others as late as 3 years from aspirate injection [17]. In the studies that do report adverse events, the most common event is mild discomfort from donor site that resolved in several days [17, 22]. Manner of reporting adverse events was extremely heterogeneous, and no accurate rate can be recorded.

Several articles included other results of interest outside of union rates and adverse events. Hernigou et al. noted that BMAC CFU count was significantly higher in the patient cohort that did successfully unite compared to patients with persistent nonunion [18]. Two studies collected patient-reported outcomes (PROs) and noted improvements in AAOS lower limb core score [21], SF-12 physical component summary [21], and VAS pain score [24]. Lastly, one study supplemented BMAC with a growth factor (rhBMP-2) and reported a 70% union rate with added growth factor compared to an 86% without BMP (Table 1) [25].

### Septic nonunion

Seven studies reported on use of BM or BMAC for the treatment of septic nonunion [11, 27–32]. Published articles ranged from 1999–2003, and all articles reported on use in adults. One of the seven articles provided Level I evidence as a prospective randomized control trial [29]. All other articles level of evidence ranged from IV to II. Three articles report on use of BM [27, 28, 32], while four report on use of BMAC [11, 29–31]. Five studies (one case report) investigated use in the tibia [11, 27, 29, 30, 32], one study described use in the femur [28], and one case report pertained to use in the humerus [31]. Four studies reported use of a scaffold, including femoral head allograft (2), iliac crest autograft, and bioactive glass. One study reported on re-injection of one patient at 4 months post-op [30]. See Table 2 for overview of studies concerning use of BM and BMAC in septic nonunion.

**Table 2:** Overview of studies utilizing bone marrow (BM) and bone marrow aspirate concentrate (BMAC) for treatment of septic nonunion

	Study size	Location	Biologic	Volume	Scaffold	Union rate	Level of evidence
Sebecic, 1999 [32]	1	Tibia	BM	150 mL	None	1	IV
Ateschrang, 2009 [27]	15	Tibia	BM	not reported	Femoral head graft cut up into chips	0.73	II
Schroter, 2016 [28]	18	Femur	Bm	not reported	Femoral head allograft	0.83	II
Hernigou, 2016 [11]	30	Tibia	BMAC	20 mL	None	0.833 at 6 mo, 1.00 at 12 mo	IV
Hernigou, 2018 [29]	80	Tibia	BMAC	20 mL	Iliac crest cancellous bone vs cancellous bone + bmac	.95 in BMC by 1 year .70 w/o BMC by 1 yaer	I
Van Vugt, 2021 [30]	5	Tibia	BMAC	6.2 mL	S53P4 bioactive glass (BonAlive)	1	III
Williams, 2023 [31]	1	Humerus	BMAC, PRP, Platelet Lysate	4 mL BMAC, 1 mL PRP, 1 mL PL	None	1	IV

All septic studies uniformly adhered to a minimum time to nonunion of 6 months, with a range of 6–244 months reported. Sebecic et al. report use of 150 mL BM, while the Ateschrang et al. and Schroter et al. do not report volumes. BMAC use ranged from 4 to 20 mL, with a similar requirement of 60–120 mL BM to achieve BMAC volume. No studies reported on use of multiple injections. A case report by Williams et al. reported on use of a mixed biologic consisting of 4 mL BMAC, 1 mL PRP, and 1 mL platelet lysate.

Prior to use of BM/BMAC, infection control was obtained with combination of antibiotics, debridement, and hardware removal when indicated. Use of external fixator vs initial fixation was determined on a case-by-case basis, and all but one study included use of post-operative antibiotics. BM studies reported union rates of 73, 83, and 100% (Table 2) [27, 28, 32]. BMAC studies reported rates of 83.3, 95, 100, and 100% (Table 2) [11, 29–31]. Mean time to union ranged from 12 weeks to 1 year. All studies reported satisfactory infection control, with 2 BM and 2 BMAC reporting 0% reinfection rates, and three studies reporting 3.33, 6.67, and 17.5% reinfection rate. In the study reporting 17.5% infection rate at a mean follow-up of 7 years, 2/40 (5%) patients received BMAC + fresh iliac crest autograft, while 12/40 (30%) patients received fresh iliac crest autograft without BMAC [29].

One manuscript was designed to study the ability to treat infection without use of antibiotics and solely through bone marrow-derived granulocytes [11]. The study included 30 tibial septic nonunion patients who failed previous surgical treatments. All patients included were noted to have history of sinus tract that resolved following < 60 days of antibiotics, isolation of bacteria, and elevated CRP. Following resolution of the sinus tract, antibiotics were stopped, and patients were then treated with ex-fix and BMAC (18 atrophic, 12 hypertrophic). 100% of these patients went onto union by 1 year post-op, and 27/30 patients had normalization of elevated CRP by 21 days post-BMAC. Two patients developed external fixator pin-site infections, while only one patient was found to have recurrence of bony infection within 10 years follow-up. It was noted that peripheral blood and fracture site granulocyte–macrophage levels were significantly lower in polytrauma patients when compared to healthy controls, while BMAC granulocyte–macrophage levels did not statistically differ from healthy controls.

Adverse event profile was overall similar when compared to use in aseptic nonunion, with a couple notable exceptions. Donor site pain was the most commonly reported adverse event, with one study reporting superficial wound infection that resolved without treatment [11]. Similar to aseptic nonunion studies, heterogeneous reporting of adverse events prevents accurate assessment of exact adverse event rates. In a study reporting on use of BMAC with bioactive glass, two patients required reoperation for either screw breakage or fistula persistence [30]. A separate study reported infection control in all patients and three patients with persistent nonunion, of which two required above knee amputation [28].

### Mixed nonunion

Five studies describe the use of BM and BMAC in a mixed patient population, consisting of both septic and aseptic nonunion [33–37]. Three of the studies included the use of BM and ranged from 1989 to 1995 while newer studies from 2014 to 2023 included the use of BMAC [36]. Level of evidence ranged from IV to III. Two studies report on use of BM in tibia, while two studies report use in a combination of long bones. Only one study did not use a scaffold [33], while all others used DBM. Table 3 outlines use of BM and BMAC in mixed-case nonunions.

**Table 3:** Overview of studies utilizing bone marrow (BM) and bone marrow aspirate concentrate (BMAC) for treatment of mixed septic and aseptic nonunion

	Study size	Location	Biologic	Volume	Scaffold	Union rate	Level of evidence
Connoly, 1989 [33]	10	Tibia	BM	150 mL	None	0.9	IV
Connoly, 1991 [34]	20 (10 infectious, 10 non-infectious)	Tibia	BM	150 mL	DBM in 3 patients with large sequestrum	0.8 with external treatment, 1.00 with internal treatment	IV
Tiedeman, 1995 [35]	39 (9 of original 48 loss to FU or without adequate data)	Clavicle, humerus, femur, tibia (only 18 patients with specific details reported)	BM	10–100 mL	DBM	0.77	IV
Scaglione, 2014 [36]	19	Humerus (2), radius (3), ulna (1), forearm (1), femur (1), tibia (1), fibula (1), metatarsal (NA—1), infected/open (4)	BMAC	20 mL aspirate prior to centrifuge	DBM	0.79	III
Canton, 2023 [37]	11	Clavicle (1), humerus (1), tibia (5), femur (4),	BMAC	Not reported	Cancellous allograft (9 patients), ringed ex-fix (2)	1	III

For mixed population studies, time to BM vs BMAC use varied from four to 36 months. BM was used at a volume of 150 mL and 10–100 mL, while one study reported centrifuging 20 mL of bone marrow aspirate into BMAC prior to mixing with 10 cc DBM.

In the mixed cohort of infected and non-infected union, fixation technique was heterogeneous, and supplementation with antibiotics and staging processes varied depending on the presence of infection both within and between studies [35, 36]. Use of adjuvant therapies such as electrical stimulation was also reported [34]. Overall, union rates were reported to be between 77 and 100% across all studies. Some subgroups within studies, such as internal fixation group in Connoly et al. 1991. study were reported to reach 100% while casting was reported to have a rate of 80% [34]. Further characterization of rates by treatment modality cannot be reported due to manuscript reporting differences.

While all studies report minor discomfort at donor site, a few other notable adverse events are highlighted. Connoly et al. are among the first to note increased donor site discomfort and blood dilution of aspirate when pulling large volumes of aspirate [33]. This study also reported on burning at injection site attributed to use of a large needle in one patient [33]. Two studies do note infection following use of BM/BMAC. Tiedeman et al. report one patient with infection following IM rod and BM + DBM, who subsequently went on to fail antibiotics and requiring hardware removal [35]. Scaglione et al. report that one aseptic nonunion patient within the cohort did develop tibial bone infection at site of BM/DBM use, requiring further intervention and debridement with Masquelet’s technique [36].

While not included in any of the groups above, one study reports on use of 50 mL BM in in an oncology setting. A major adverse event of heterotopic ossification at site of injection was reported, requiring surgical excision and radiation.

- *Question 2: Does recent literature highlight a potential mechanism of action?*

In addition to the above 41 articles screened, additional articles discuss potential mechanism of action for BM/BMAC [38–40]. Early studies from the late 1990’s and early 2000’s hypothesize that marrow progenitor cells may differentiate into bone and cartilage under the influence of cytokines and the transforming growth factor beta super family [41]. In vitro studies demonstrated the mesenchymal stem cell differentiation to terminal hypertrophic chondrocyte with an increase in alkaline phosphatase signifying potential mineralization, while in vivo chimeric mouse studies showed fluorescent-labeled mesenchymal stem cells predominately in the fracture callus [42]. Contrary to this, a 2006 study reported that actual bone marrow-derived cells may not directly participate in fracture healing, but instead stimulates bone repair through inducing bone and cartilage differentiation of other cell sources [43].

By 2011, research focus shifted toward a more holistic view of fracture healing, with the diamond concept outlining a need for biomaterial scaffold, cell biology, growth factors, and a mechanically stable environment [44]. Especially with BM containing between 0.001 and 0.01% MSCs, studies began to report on the importance of cytokine and growth factor signaling in overall fracture response [45]. Specifically for the nonunion model, a 2013 study suggested that the microenvironment in atrophic nonunion impedes endogenous progenitor cells, and that biologics can reactivate these endogenous cells to stimulate healing.^1^ This paracrine model was further supported in 2021 [46].

## Discussion

This study answers two major questions. (1) BM and BMAC in combination with other treatments seem to have satisfactory union rates. Study heterogeneity and a lack of level I prevent understanding outcomes of BM/BMAC alone as well as the relative contribution of BM/BMAC vs other gold standard treatments when co-administered. (2) BM and BMAC mechanism of action is more likely due to secretory effect and host immunomodulatory response, and less likely due to direct cellular seeding.

When compared to the literature, two major agreements are seen. Firstly, previous review notes iliac crest autograft to have a successful union rate of 87–100% when used for nonunion [47]. Allograft represents an additional treatment option, but current literature highlights lower union rates and higher infection rates when compared to autograft [5, 6]. While some BM/BMAC articles report lower union rates than autograft, the majority (18/24) report a union rate > 80%, comparable to autograft. It is important to note some studies do report union rates as late as 3 years post-injection, which more than likely cannot be attributed to use BM/BMAC [17]. Other contemporary literature reports on use of BM/BMAC in a similar fashion. [21] In particular, the highest rates of union were seen with use of BMAC in septic nonunion, with union rates of 83.3–100%. In these septic cases, supplementation of BMAC was seen to reduce the rate of reinfection in iliac crest autograft by 25% (*P* = 0.009) [29]. Use of BMAC was also seen to successfully treat septic nonunion even with cessation of antibiotics [11]. This was attributed to normalization of granulocyte–macrophage levels with concentration of bone marrow aspirate [11].

In terms of mechanism, most recent literature seem to support a secretory role of BM and BMAC [38, 45, 46]. While early studies focused on direct proliferative potential of BM/BMAC, newer studies offer conflicting evidence [42, 43]. When used in vivo, intravenous MSCs are not detectable within 24 h, while intra-articular MSCs are not detectable within 1 month [48, 49]. Most contemporary literature recognizes progenitor cell secretory function, or secretome, as the predominate mechanism of action for MSCs with some potential importance attributed to bone morphogenic protein-2 (BMP-2) host signaling response in fracture healing [50–53].

This review is not without its limitations. As described in other recent reviews, the literature pertaining to use of bone marrow in long-bone nonunion is extremely heterogeneous [54]. Unfortunately, this review highlights the same findings. The current literature is extremely heterogeneous with regard to study design and characteristics, patient population medical and surgical history, concomitant fixation, and types of scaffolds. With the use of BM and BMAC, this study highlights 9 different types of scaffolds used in a wide variety of manners [16, 17, 20, 25, 27–30, 36]. Fixation construct varied from use of casting/splinting, ex-fix, IM rods, IM nails, plating, and k-wires. Given this wide heterogeneity, it is difficult to determine the relative contribution of fixation construct vs scaffold vs BM/BMAC. These inconsistencies prevented the feasibility of completing a meta-analysis from the data cited in this study. Secondly, this review highlights a relative lack in understanding of BM/BMAC mechanism of action, despite over 30 years of use. Of the studies reported in this review, a minority directly addresses the mechanism of action for percutaneous BM/BMAC injection. While in vivo function and pre-clinical trials are certainly important, these findings are not guaranteed to carry over into in vivo percutaneous injection. Lastly, this review only includes three modern studies completed since 2020 and only one study with Level I evidence. Without high-quality modern studies, no strong conclusions can be made. Nonetheless, the findings in this study and in recent literature allow for several broad conclusions.

## Conclusion

The current literature pertaining to use of BM/BMAC for nonunion is extremely heterogeneous in terms of patient population and concomitant treatment modalities. While results are promising for use of BM/BMAC with other gold standard treatment methodologies, the literature requires additional Level I studies are needed to clarify the impact of role BM/BMAC in treating nonunion when used alone and in combination with other modalities.
